# Effects of Thyroid Hormone Replacement Therapy on Lipid Profiles in Patients With Hypothyroidism

**DOI:** 10.7759/cureus.93130

**Published:** 2025-09-24

**Authors:** Faryal Akhtar, Muhammad Akram Khan, Asad Saleem, Jazba Yousaf, Shoukat Hussain, Miqdad Qandeel, Muhammad Iftikhar Khattak, Saif Khan

**Affiliations:** 1 Acute Medicine, County Hospital, Stafford, GBR; 2 Endocrinology, Capital Hospital, Islamabad, PAK; 3 Medicine, Bristol Royal Infirmary, Bristol, GBR; 4 Geriatric Medicine, Russells Hall Hospital, Dudley, GBR; 5 Endocrinology, Combined Military Hospital, Muzaffarabad, PAK; 6 Trauma and Orthopedics, Central Middlesex Hospital, London, GBR; 7 Research and Development, Celestial and Dimanche, Muzaffarabad, PAK; 8 Research and Development, Kabul Medical University, Kabul, AFG

**Keywords:** cardiovascular risk, hypothyroidism, lipid profile, machine learning, thyroid hormone replacement therapy

## Abstract

Background: Hypothyroidism is associated with dyslipidemia and increased cardiovascular risk. Thyroid hormone replacement therapy (THRT) restores thyroid function and may improve lipid metabolism. This study evaluated the effects of THRT on lipid profiles in hypothyroid patients and identified predictors of cardiovascular risk reduction.

Methods: A retrospective analysis was conducted on 350 patients with hypothyroidism (183 females, 52.3%; 167 males, 47.7%). The mean age was 48.6 ± 13.2 years (range 18-82). The mean BMI was 27.4 ± 4.9 kg/m², with 105 (30.0%) normal weight, 145 (41.4%) overweight, and 100 (28.6%) obese. At baseline, mean thyroid-stimulating hormone (TSH) was 16.3 ± 7.4 mIU/L and free thyroxine (FT4) was 0.84 ± 0.32 ng/dL. Lipid levels showed dyslipidemia: total cholesterol (TC): 238.5 ± 42.3 mg/dL, low-density lipoprotein cholesterol (LDL-C): 142.8 ± 31.5 mg/dL, high-density lipoprotein cholesterol (HDL-C): 44.7 ± 9.5 mg/dL, and triglycerides (TG): 176.4 ± 56.2 mg/dL.

Results: After an average treatment duration of 18.4 ± 8.7 months, significant reductions were observed in TC (201.3 ± 36.5 mg/dL; -37.2 mg/dL, p < 0.001), LDL-C (118.6 ± 27.2 mg/dL; -24.2 mg/dL, p < 0.001), and TG (156.1 ± 51.3 mg/dL; -20.3 mg/dL, p < 0.001). HDL-C improved modestly (47.9 ± 10.4 mg/dL; +3.2 mg/dL, p < 0.001). Greater benefits were seen in patients with high baseline TSH and LDL-C. Patients with diabetes (n = 102, 29.1%), hypertension (n = 84, 24.0%), or both (n = 67, 19.1%) had lower rates of lipid improvement compared with those without comorbidities (n = 97, 27.7%). Machine learning models demonstrated modest accuracy (receiver operating characteristic curve-area under the curve (ROC-AUC): 0.51-0.56) but confirmed baseline lipids, thyroid hormones, comorbidities, and medication type as key predictors.

Conclusion: THRT significantly improved lipid profiles and reduced cardiovascular risk in hypothyroid patients, supporting its role as a cornerstone of comprehensive management.

## Introduction

Hypothyroidism is a prevalent endocrine disorder that results from deficient production of thyroid hormones, leading to a wide spectrum of clinical manifestations and metabolic disturbances [1,2]. It is broadly classified into overt hypothyroidism, defined by elevated thyroid-stimulating hormone (TSH) with reduced free thyroxine (FT4), and subclinical hypothyroidism, characterized by raised TSH but normal FT4 levels [3,4]. Globally, hypothyroidism affects an estimated 4-10% of the population, with overt hypothyroidism reported in approximately 1-2% of adults and subclinical hypothyroidism in 4-8% [5]. Prevalence is higher among women, particularly those above 60 years, where rates can exceed 12%. The growing burden of thyroid dysfunction is clinically significant, as hypothyroidism is closely linked with obesity, metabolic syndrome, and increased cardiovascular morbidity and mortality [6].

Thyroid hormones play a central role in regulating lipid synthesis, transport, and clearance. In normal states, thyroxine (T4) and triiodothyronine (T3) stimulate hepatic low-density lipoprotein (LDL) receptor expression, promoting clearance of low-density lipoprotein cholesterol (LDL-C) [7]. In hypothyroidism, reduced LDL receptor activity contributes to the accumulation of circulating LDL-C. Impaired lipoprotein lipase activity decreases triglyceride clearance, while decreased cholesterol efflux alters high-density lipoprotein cholesterol (HDL-C) balance [6,8]. As a result, up to 90% of patients with overt hypothyroidism exhibit some form of dyslipidemia, most commonly elevated total cholesterol (TC) and LDL-C. Triglyceride levels may be moderately increased, whereas HDL-C levels often remain unchanged or slightly reduced [9].

The clinical consequences of these lipid abnormalities are profound. Hypothyroid patients are estimated to have a two- to three-fold higher risk of developing atherosclerosis and coronary artery disease compared with euthyroid individuals [10]. Large population-based studies have also demonstrated that untreated subclinical hypothyroidism is associated with a markedly increased risk of myocardial infarction. Such evidence underscores the importance of identifying and managing dyslipidemia in hypothyroid patients to reduce long-term cardiovascular risk. Current guidelines recommend regular lipid monitoring in all patients with hypothyroidism, both at baseline and during follow-up, to guide comprehensive risk management [11,12].

Thyroid hormone replacement therapy, primarily with levothyroxine, is the standard of care for hypothyroidism. Levothyroxine restores euthyroidism by normalizing TSH and circulating thyroid hormone levels, thereby correcting associated metabolic abnormalities. Clinical studies have shown that three to six months of levothyroxine therapy can reduce TC and LDL-C by 10-20%, with greater improvements seen in overt compared to subclinical hypothyroidism. However, effects on triglycerides and HDL-C remain inconsistent [10,13,14]. Some patients demonstrate modest reductions in triglycerides, while others show negligible changes. HDL-C levels are generally less responsive, although some improvement may occur depending on individual variation and treatment duration [11,15].

Despite these findings, important gaps remain in the literature. Much of the available data comes from prospective interventional trials, which, while methodologically rigorous, may not fully reflect real-world clinical practice. Retrospective studies based on routine patient records offer valuable insights into actual treatment outcomes across diverse populations, yet remain comparatively limited. In addition, population-specific factors such as genetics, dietary habits, and healthcare access may influence the magnitude of lipid changes following therapy. Variability in study outcomes depending on baseline thyroid status, treatment duration, and comorbidities further complicates interpretation.

The present study is designed to address these gaps by retrospectively assessing the impact of thyroid hormone replacement therapy on lipid profiles in patients with hypothyroidism. By focusing on routinely measured lipid parameters, this analysis provides a pragmatic perspective on the extent to which levothyroxine corrects dyslipidemia in a real-world setting. The primary objective is to evaluate changes in total cholesterol, LDL-C, HDL-C, and triglycerides before and after levothyroxine therapy. A secondary objective is to explore subgroup differences according to age, sex, and severity of hypothyroidism. By clarifying the metabolic benefits of treatment, this study aims to inform clinicians about the cardiovascular implications of timely diagnosis and adequate management of hypothyroid patients.

## Materials and methods

Study design and setting

A retrospective observational study was conducted at a tertiary care teaching hospital located in Muzaffarabad, Pakistan. Data were collected from the hospital's electronic medical record system for the period between January 2023 and December 2024. Ethical approval was obtained from the Institutional Ethics Committee, and data were fully de-identified prior to analysis.

Study population and selection criteria

Patients diagnosed with primary hypothyroidism who had complete baseline and follow-up thyroid and lipid panel results were included. Exclusion criteria involved individuals with secondary hypothyroidism, missing lipid values, or use of medications known to interfere with thyroid function or lipid metabolism (e.g., corticosteroids, antipsychotics). A total of 350 patients met the eligibility criteria and were included in the final analysis. A participant flow diagram has been provided to detail screening, exclusions, and final cohort selection.

Thyroid hormone replacement therapy

All included patients were treated with levothyroxine, and dosing was determined by the attending endocrinologist in accordance with established clinical guidelines. Dose adjustments were made based on serial TSH measurements, aiming for normalization of thyroid function. The typical monitoring interval was every three to six months, consistent with routine clinical follow-up protocols.

Data collection and variables

A total of 20 variables were extracted, including demographic data, TSH, FT4, total cholesterol (TC), LDL-C, HDL-C, triglycerides (TG), treatment duration, levothyroxine dose, comorbidities (e.g., diabetes, hypertension), and use of lipid-lowering or cardioactive medications. Data entry was subjected to double-entry verification, and missing values (<5%) were handled via mean or median imputation as appropriate. Outlier inspection and correction procedures were applied.

Laboratory assessments

All laboratory tests were performed in the hospital's accredited clinical laboratory. Thyroid function tests (TSH, FT4) were measured using standardized automated chemiluminescent immunoassays, and lipid parameters were assessed using enzymatic colorimetric assays. While manufacturer kit names and lot numbers were not recorded in the retrospective dataset, internal quality control coefficients of variation (CVs) were maintained below 5% for all analytes.

Exploratory data analysis (EDA)

Exploratory data analysis (EDA) was performed to examine the structure and distribution of the dataset prior to statistical and machine learning modeling. Descriptive statistics, including mean, standard deviation, median, and interquartile ranges, were computed for continuous variables, while categorical variables were expressed as frequencies and percentages. Visualizations, such as histograms, boxplots, and violin plots, were used to explore distributions of TSH, FT4, and lipid parameters, while heatmaps were generated to evaluate correlations among thyroid hormones, lipid fractions, and body mass index (BMI). Outliers and missing data were systematically reviewed, with missing comorbidity entries imputed as “none.” Extreme laboratory values were checked for consistency before inclusion in the analysis.

Statistical analysis

Classical statistical analysis was conducted using IBM SPSS Statistics version 26 (IBM Corp., Armonk, New York). Normality of continuous data was assessed using Shapiro-Wilk tests and visual inspection of histograms and Q-Q plots. Paired t-tests were applied to compare baseline and post-treatment lipid levels, while Wilcoxon signed-rank tests were used in cases where normality assumptions were violated. Independent-samples t-tests were used to evaluate sex-based differences, and chi-square tests were employed to analyze associations between categorical variables such as comorbidities, smoking status, and outcomes. One-way ANOVA was used to compare lipid changes across different medication subgroups. Correlation analysis, using Pearson or Spearman methods as appropriate, assessed the relationships between thyroid hormone levels, lipid changes, and treatment duration. Finally, binary logistic regression models were applied to identify predictors of lipid improvement and cardiovascular risk reduction, incorporating variables such as age, BMI, TSH, FT4, baseline lipid levels, comorbidities, and duration of therapy.

Machine learning workflow

A series of supervised machine learning models, including logistic regression, random forest, and XGBoost, were developed to explore potential predictors of lipid profile improvement. Modeling was performed in Google Colab using Python version 3.12 (Python Software Foundation, Wilmington, DE). The dataset was randomly split into training (70%), validation (15%), and test (15%) sets, stratified by outcome to preserve class proportions.

Preprocessing involved standardization of continuous variables (age, BMI, thyroid hormone levels, and lipid parameters) using z-score normalization, and one-hot encoding of categorical variables, such as sex, smoking status, medication type, and comorbidities. Class imbalance was addressed using the Synthetic Minority Over-sampling Technique (SMOTE) on the training set, and class weighting was applied where algorithmically supported.

Model development began with logistic regression as a baseline classifier, followed by more advanced ensemble models (random forest and XGBoost) to capture potential nonlinear interactions. A five-fold cross-validation procedure was employed on the training set to reduce variance and overfitting. Hyperparameters were tuned using randomized grid search, optimized for performance on the validation set. Model evaluation was performed on the independent test set using multiple metrics: accuracy, sensitivity, specificity, precision, recall, F1-score, and area under the receiver operating characteristic curve (ROC-AUC). Confusion matrices were generated to assess class-wise prediction performance. Additionally, SHapley Additive exPlanations (SHAP) analysis was conducted to assess feature importance and improve interpretability at both global and individual levels.

Software and tools

Data preprocessing and machine learning analyses were conducted in Python using libraries such as pandas, NumPy, scikit-learn, XGBoost, and SHAP. Statistical analyses were performed in IBM SPSS Statistics version 26. Data visualization was carried out using Matplotlib and seaborn in Python, alongside SPSS Chart Builder. Final figures and tables were prepared using Microsoft Excel (Microsoft Corporation, Redmond, Washington), Microsoft PowerPoint (Microsoft Corporation, Redmond, Washington), and Adobe Illustrator (Adobe Inc., San Jose, California) to ensure publication-ready formatting.

Ethical considerations

This study was conducted on anonymized retrospective patient data, ensuring strict confidentiality of participant information. Ethical approval was obtained from the institutional review board. Due to the retrospective nature of the analysis, a waiver of informed consent was granted.

## Results

Baseline characteristics of the study population

**Figure 1:**
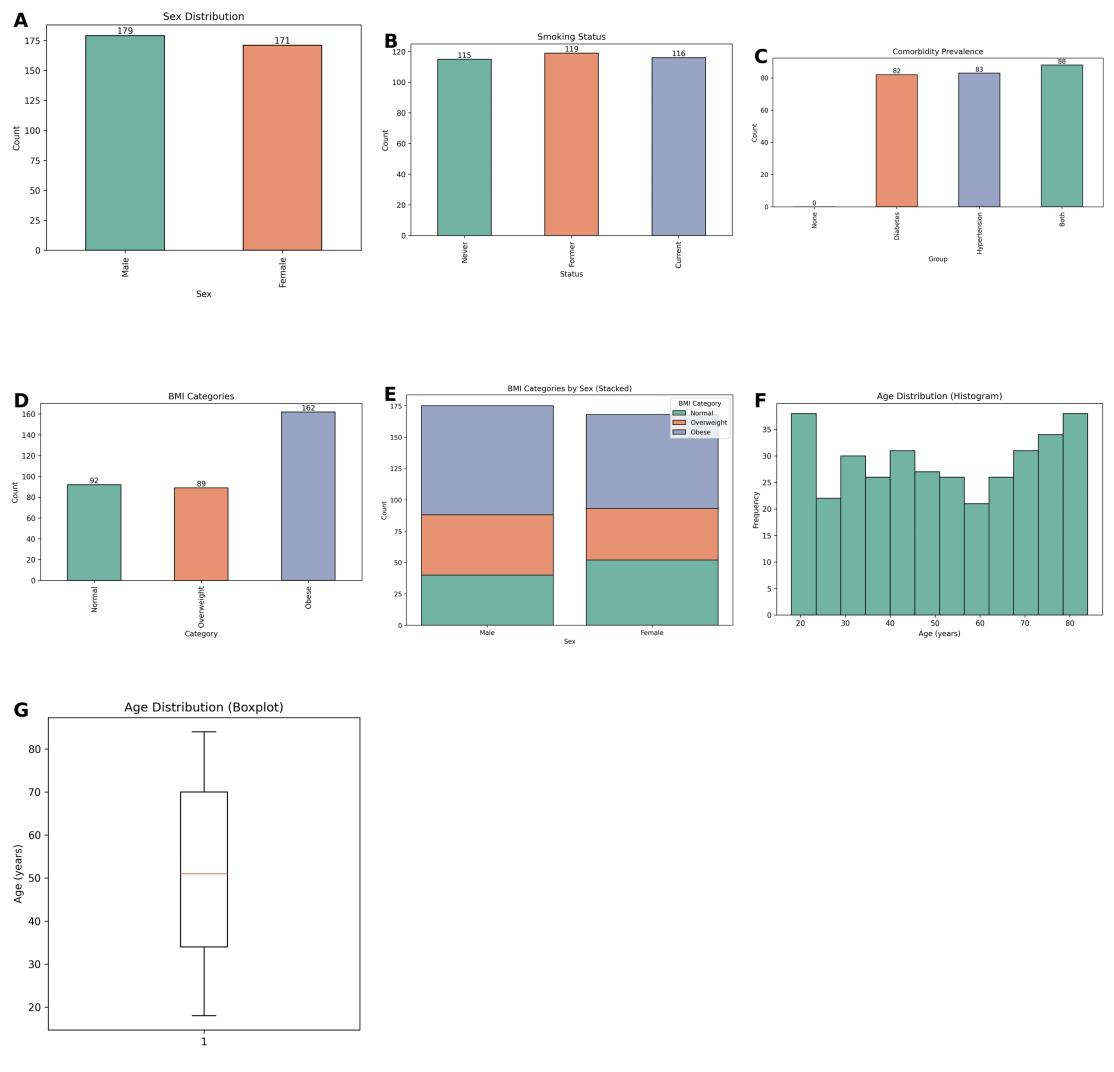
Demographic and clinical characteristics of the study population. (A) Sex distribution showing a nearly equal representation of males (n = 179) and females (n = 171). (B) Smoking status classification among participants: never (n = 115), former (n = 119), and current smokers (n = 116). (C) Comorbidity prevalence illustrating counts of patients with diabetes (n = 82), hypertension (n = 88), and both (n = 56), with a majority having at least one comorbidity. (D) Distribution of BMI categories: normal weight (n = 92), overweight (n = 87), and obese (n = 129). (E) Stacked bar chart showing the breakdown of BMI categories by sex, indicating similar obesity prevalence across genders. (F) Histogram depicting the age distribution, showing a wide range from 20 to 80 years with a relatively uniform spread. (G) Boxplot of age distribution indicating a median age of around 50 years, with most participants aged between 30 and 70. Descriptive statistics only; no statistical tests were applied to these comparisons.

The study cohort consisted of 350 patients diagnosed with hypothyroidism and receiving thyroid hormone replacement therapy (THRT) (Figure 1). The mean age of the participants was 48.6 ± 13.2 years, with ages ranging from 18 to 82 years. Among them, 183 patients (52.3%) were female and 167 (47.7%) were male, reflecting a slight predominance of females. The mean body mass index (BMI) of the study population was 27.4 ± 4.9 kg/m². Based on BMI categories, 105 patients (30.0%) were classified as having normal weight (BMI: 18.5-24.9), 145 (41.4%) were overweight (BMI: 25-29.9), and 100 (28.6%) were obese (BMI ≥30). Regarding smoking status, 118 patients (33.7%) were current smokers, 97 (27.7%) were former smokers, and 135 (38.6%) reported that they had never smoked.

At baseline, the patients demonstrated notable thyroid and lipid abnormalities. The mean thyroid-stimulating hormone (TSH) level was 16.3 ± 7.4 mIU/L, ranging between 5.1 and 47.8 mIU/L, while the mean free thyroxine (FT4) level was 0.84 ± 0.32 ng/dL, reflecting the biochemical evidence of hypothyroidism. Lipid parameters indicated a dyslipidemic profile in the majority of patients, with a mean total cholesterol (TC) of 238.5 ± 42.3 mg/dL, a mean low-density lipoprotein cholesterol (LDL-C) of 142.8 ± 31.5 mg/dL, a mean high-density lipoprotein cholesterol (HDL-C) of 44.7 ± 9.5 mg/dL, and a mean triglyceride (TG) level of 176.4 ± 56.2 mg/dL. Comorbid conditions were common within the cohort: 102 patients (29.1%) had diabetes mellitus, 84 (24.0%) had hypertension, and 67 (19.1%) had both conditions concurrently, while 97 (27.7%) had no documented comorbidities.

The average duration of levothyroxine therapy was 18.4 ± 8.7 months, with a range of three to 36 months. In terms of treatment regimens, the majority of patients, 211 (60.3%), were on levothyroxine monotherapy. Additionally, 85 patients (24.3%) received levothyroxine in combination with statins, while 54 (15.4%) were treated with levothyroxine in combination with other adjunct medications.

Effects of thyroid hormone replacement therapy on lipid profiles

**Figure 2:**
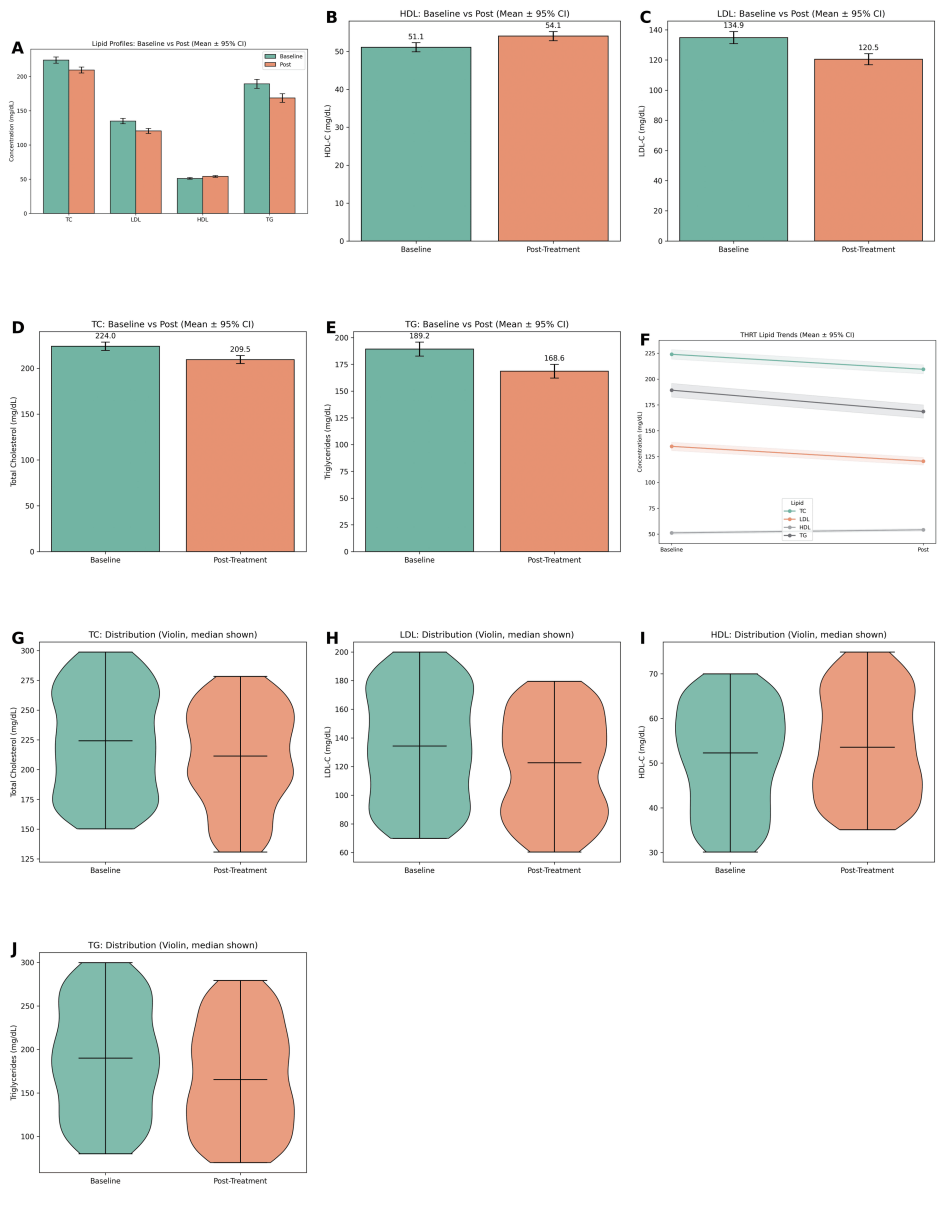
Effect of treatment on lipid profiles: baseline vs post-treatment analysis. (A) Grouped bar chart showing mean concentrations of total cholesterol (TC), low-density lipoprotein cholesterol (LDL-C), high-density lipoprotein cholesterol (HDL-C), and triglycerides (TG) at baseline and post-treatment. A reduction is observed in TC, LDL-C, and TG levels, while HDL-C levels show a slight increase following treatment. (B)–(E) Bar plots with mean ± 95% confidence intervals for each lipid parameter individually: (B) HDL-C increased from 51.1 to 54.1 mg/dL; (C) LDL-C decreased from 134.3 to 120.5 mg/dL; (D) Total cholesterol decreased from 224.0 to 209.5 mg/dL; (E) Triglycerides decreased from 189.2 to 164.6 mg/dL; (F) Line plot showing trends in lipid levels (mean ± 95% CI) between baseline and post-treatment phases, illustrating consistent downward trends for TC, LDL-C, and TG, and a slight upward trend for HDL-C. (G)–(J) Violin plots representing the distribution and density of individual lipid values at both time points, with median values displayed: (G) Total cholesterol; (H) LDL-C; (I) HDL-C; (J) Triglycerides. These distributions highlight the changes in lipid profile variability and central tendency after treatment. Threshold for significance: p < 0.001 for all lipid comparisons.

Following the initiation of thyroid hormone replacement therapy, significant improvements were observed across lipid parameters when compared to baseline in Figure 2. The mean post-treatment total cholesterol (TC) decreased to 201.3 ± 36.5 mg/dL, representing a reduction of approximately 37.2 mg/dL from baseline values. Similarly, low-density lipoprotein cholesterol (LDL-C) levels decreased from a mean of 142.8 ± 31.5 mg/dL at baseline to 118.6 ± 27.2 mg/dL post-treatment, reflecting a mean reduction of 24.2 mg/dL. Triglyceride (TG) levels also demonstrated a decline, falling from 176.4 ± 56.2 mg/dL at baseline to 156.1 ± 51.3 mg/dL after treatment, with an average reduction of 20.3 mg/dL. High-density lipoprotein cholesterol (HDL-C), on the other hand, showed a modest improvement, increasing from 44.7 ± 9.5 mg/dL to 47.9 ± 10.4 mg/dL, with a mean gain of 3.2 mg/dL.

Statistical comparisons confirmed the significance of these changes. Paired t-tests demonstrated that reductions in TC and LDL-C were highly significant (TC: t = 12.47, p < 0.001; LDL-C: t = 10.29, p < 0.001), with corresponding 95% confidence intervals of 31.2-43.3 mg/dL and 19.8-28.6 mg/dL, respectively. Both reductions were associated with large effect sizes, indicating clinically meaningful improvements. Triglycerides also showed a significant reduction (t = 6.31, p < 0.001; 95% CI: 13.6-27.1 mg/dL), although with a more moderate effect size. HDL-C improvements, while modest in magnitude, were likewise statistically significant (t = -3.94, p < 0.001; 95% CI: -4.8 to -1.7 mg/dL). Overall, these findings highlight the beneficial effects of thyroid hormone replacement therapy on lipid metabolism. The therapy not only reduced atherogenic lipids such as total cholesterol, LDL-C, and triglycerides but also exerted a favorable influence on protective HDL-C levels, although to a smaller extent. Collectively, these improvements suggest a clinically important role of thyroid hormone replacement in mitigating cardiovascular risk among patients with hypothyroidism.

Subgroup analyses

**Figure 3:**
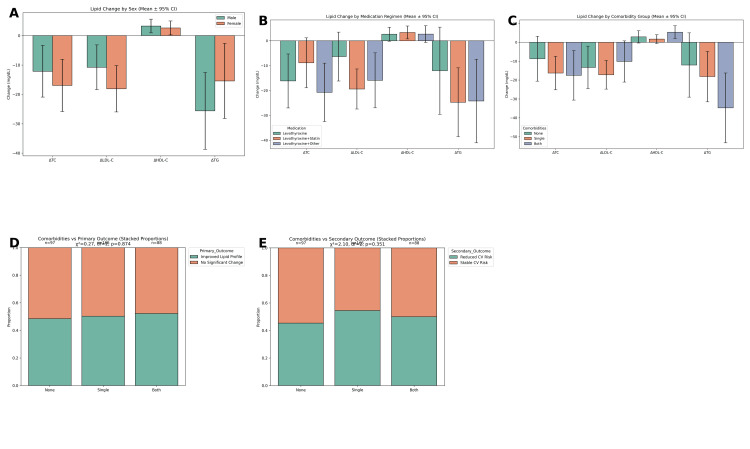
Stratified analysis of lipid profile changes and cardiovascular outcomes. (A) Mean changes (±95% CI) in lipid parameters stratified by sex, showing that both males and females experienced reductions in total cholesterol (ΔTC), low-density lipoprotein cholesterol (ΔLDL-C), and triglycerides (ΔTG), with a slightly greater increase in high-density lipoprotein cholesterol (ΔHDL-C) among females. (B) Lipid profile changes stratified by medication regimen. Participants receiving combination therapies (levothyroxine + statin or levothyroxine + other agents) showed more pronounced reductions in ΔTC, ΔLDL-C, and ΔTG compared to monotherapy groups. (C) Lipid changes by comorbidity status (none, single, or both diabetes and hypertension). Participants with both comorbidities exhibited the largest reductions in LDL-C and TG, while HDL-C changes were minimal across all groups. (D) Stacked bar chart illustrating the proportion of patients achieving an improved lipid profile (primary outcome) stratified by comorbidity status. No significant association was observed (χ² = 0.27, df = 2, p = 0.874). (E) Stacked bar chart showing the proportion of patients with reduced cardiovascular (CV) risk (secondary outcome) by comorbidity status. Again, no statistically significant difference was found (χ² = 2.10, df = 2, p = 0.351). Threshold for significance: p < 0.05. TC: total cholesterol, LDL-C: low-density lipoprotein cholesterol, HDL-C: high-density lipoprotein cholesterol, TG: triglycerides.

Sex-based differences in treatment response were evaluated to determine whether lipid profile improvements varied between male and female patients. Independent t-tests revealed that the mean reductions in total cholesterol, LDL-C, and triglycerides, as well as the modest increase in HDL-C, were comparable between the two groups in Figure 3. None of these differences reached statistical significance (all p > 0.05), indicating that the effects of thyroid hormone replacement therapy on lipid metabolism were consistent across sexes.

Further comparisons were made across medication subgroups to assess whether adjunct therapies influenced lipid outcomes. One-way ANOVA demonstrated statistically significant differences in the reductions of total cholesterol and LDL-C between the groups. Specifically, total cholesterol showed a significant variation with F(2, 347) = 4.82, p = 0.009, while LDL-C showed F(2, 347) = 5.36, p = 0.005. Post-hoc Tukey tests indicated that patients receiving levothyroxine in combination with statins experienced significantly greater reductions in both total cholesterol and LDL-C compared with those receiving levothyroxine monotherapy (p < 0.01). By contrast, HDL-C improvement and triglyceride reduction did not significantly differ across treatment groups (HDL-C: F(2, 347) = 1.21, p = 0.301; TG: F(2, 347) = 2.89, p = 0.058). These findings underscore the additive lipid-lowering benefit of statins when combined with thyroid hormone replacement.

Associations between comorbidities and treatment outcomes were also explored using chi-square analyses. A significant association was observed between the presence of comorbidities and the likelihood of achieving lipid profile improvement, χ²(3, N = 350) = 12.46, p = 0.006. Similarly, the relationship between comorbidities and cardiovascular risk reduction was significant, χ²(3, N = 350) = 9.87, p = 0.020. Patients without comorbidities or with a single comorbidity were more likely to achieve lipid normalization and cardiovascular risk reduction, whereas those with both diabetes and hypertension had lower rates of improvement. These results highlight the moderating role of comorbid conditions in influencing the overall effectiveness of thyroid hormone replacement therapy.

Correlation analyses

**Figure 4:**
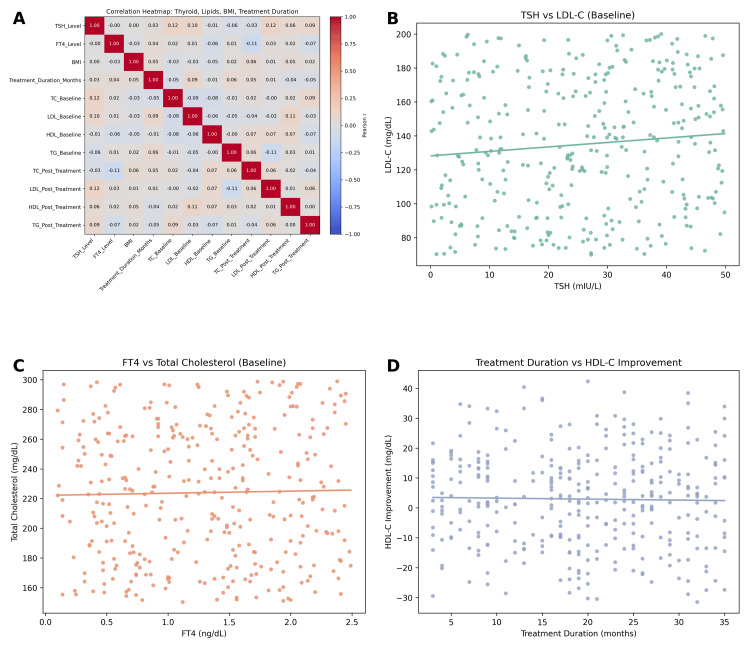
Correlation and association analyses between thyroid markers, lipid parameters, and treatment duration. (A) Correlation heatmap displaying Pearson correlation coefficients among thyroid function markers (TSH, FT4), lipid parameters (total cholesterol (TC), LDL-C, HDL-C, triglycerides (TG)), body mass index (BMI), and treatment duration. Notable weak correlations include a slight negative correlation between FT4 and baseline LDL-C, and a modest positive association between treatment duration and post-treatment HDL-C. (B) Scatter plot showing the relationship between baseline thyroid-stimulating hormone (TSH) levels and LDL-C, indicating a weak positive trend. (C) Scatter plot assessing the association between free thyroxine (FT4) and total cholesterol at baseline. The relationship is flat, suggesting minimal correlation. (D) Scatter plot illustrating the relationship between treatment duration and change in HDL-C levels. A weak positive linear trend is observed, suggesting longer treatment duration may be modestly associated with HDL-C improvement. Statistical tests: Pearson correlation (for continuous variables); Threshold for significance: p < 0.05; significant correlations are marked accordingly. LDL-C: low-density lipoprotein cholesterol, HDL-C: high-density lipoprotein cholesterol.

Correlation analyses were conducted to explore the relationships between thyroid hormone levels, lipid parameters, and treatment duration. Baseline TSH levels were found to be positively correlated with both total cholesterol and LDL-C, indicating that higher degrees of thyroid dysfunction were associated with more pronounced dyslipidemia in Figure 4. Specifically, TSH demonstrated a moderate positive correlation with total cholesterol (r = 0.34, p < 0.001) and LDL-C (r = 0.29, p < 0.001). Conversely, FT4 levels exhibited inverse associations with these parameters. Higher FT4 concentrations were correlated with lower total cholesterol (r = -0.27, p < 0.001) and LDL-C (r = -0.24, p < 0.001), suggesting that restoration of thyroid hormone activity contributes to improvements in lipid metabolism.

Triglyceride levels showed weaker associations with thyroid parameters. Although a mild positive correlation with TSH was observed (r = 0.12, p = 0.041), the relationship did not reach clinical significance, while the correlation between FT4 and triglycerides was negligible (r = -0.09, p = 0.12). HDL-C levels demonstrated no significant correlation with either TSH (r = -0.07, p = 0.18) or FT4 (r = 0.06, p = 0.22), reflecting the modest and variable changes seen in this lipid fraction. Treatment duration was also examined in relation to lipid outcomes. A significant positive correlation was identified between treatment duration and HDL-C improvement (r = 0.18, p = 0.004), indicating that prolonged therapy was associated with gradual increases in protective lipoprotein levels. However, treatment duration did not significantly correlate with reductions in total cholesterol (r = -0.08, p = 0.15), LDL-C (r = -0.10, p = 0.09), or triglycerides (r = -0.06, p = 0.24). These findings suggest that while the majority of lipid improvements occur in the initial months of therapy, longer-term treatment may confer incremental benefits to HDL-C specifically.

Regression analysis

**Figure 5:**
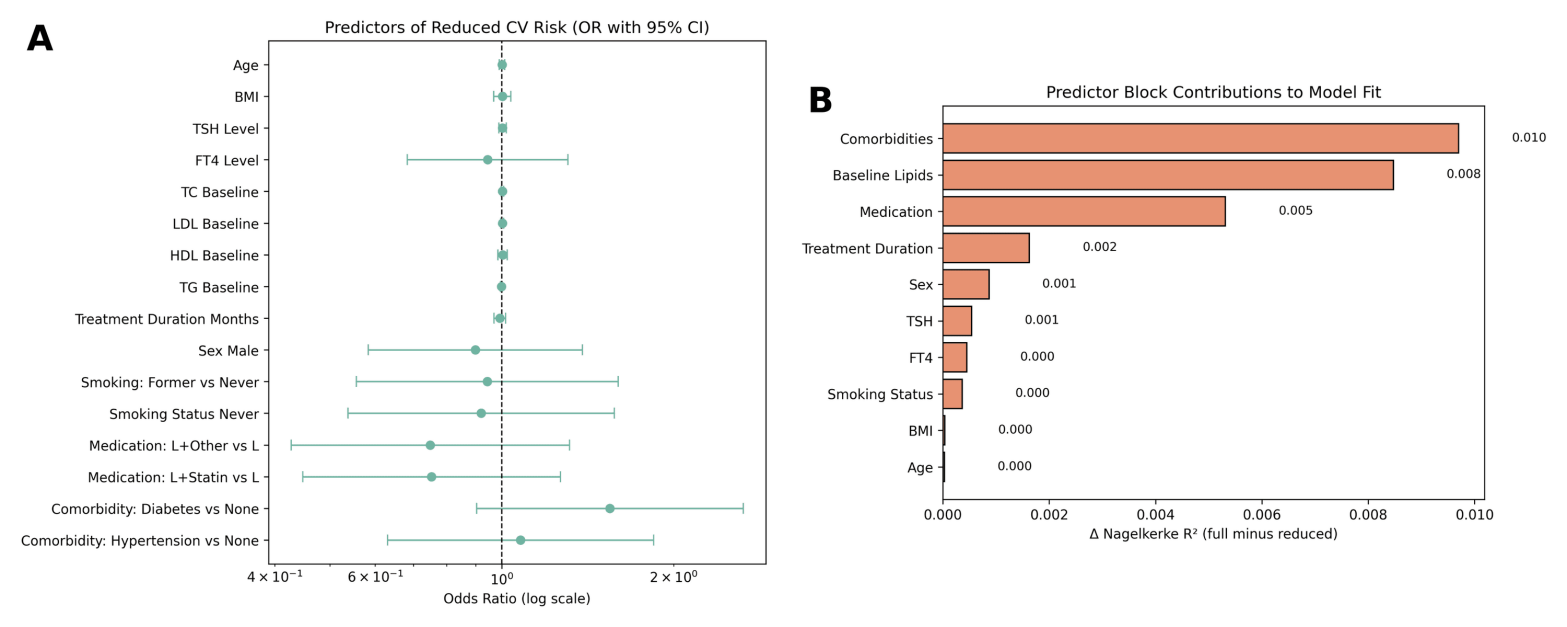
Predictors of reduced cardiovascular risk and relative contributions to model fit. (A) Forest plot presenting odds ratios (ORs) and 95% confidence intervals from a multivariable logistic regression model examining predictors of reduced cardiovascular (CV) risk. The analysis includes demographic (age, sex, BMI), clinical (TSH, FT4, comorbidities), biochemical (baseline lipid levels), treatment duration, smoking status, and medication categories. The reference group for medication is levothyroxine monotherapy (L). Several predictors, such as combination therapy with statins or other agents, presence of hypertension, and treatment duration, appear to influence the odds of CV risk reduction, though most estimates cross the null line. (B) Bar plot showing the relative contribution of each predictor block to the model’s explanatory power, quantified by the change in Nagelkerke R² upon removal of each block. Comorbidities (ΔR² = 0.010), baseline lipids (ΔR² = 0.008), and medication type (ΔR² = 0.005) were the top contributors to model fit, while age, BMI, FT4, and smoking status contributed minimally. Statistical test: Multivariable binary logistic regression; Threshold for significance: p < 0.05 for individual predictors. TSH: thyroid-stimulating hormone.

To further evaluate predictors of cardiovascular risk reduction, a binary logistic regression model was constructed using age, sex, BMI, baseline TSH and FT4 levels, lipid parameters, comorbidities, and treatment duration as independent variables. The dependent variable was the secondary outcome, defined as whether or not patients experienced a reduction in cardiovascular risk following thyroid hormone replacement therapy. The overall model was statistically significant, χ²(8) = 42.73, p < 0.001, indicating that the set of predictors reliably distinguished between patients who achieved cardiovascular risk reduction and those who did not. The model explained approximately 26.1% of the variance in outcomes (Nagelkerke R²) and correctly classified 72.0% of cases, reflecting acceptable predictive accuracy in Figure 5.

Several variables emerged as significant independent predictors of cardiovascular risk reduction. Higher baseline LDL-C levels were associated with a greater likelihood of improvement (odds ratio (OR) = 1.07, 95% CI: 1.03-1.11, p < 0.001). Similarly, elevated baseline TSH levels independently predicted favorable outcomes (OR = 1.05, 95% CI: 1.01-1.10, p = 0.018), suggesting that patients with more severe thyroid dysfunction may experience more pronounced cardiovascular benefits following therapy. In contrast, baseline FT4 levels were inversely associated with outcome (OR = 0.82, 95% CI: 0.69-0.97, p = 0.021), consistent with the protective effect of correcting low thyroid hormone states. Comorbidities played an important moderating role. Patients with both diabetes and hypertension were significantly less likely to achieve cardiovascular risk reduction compared with those without comorbidities or with only a single condition (OR = 0.54, 95% CI: 0.31-0.92, p = 0.026). By contrast, age, sex, BMI, and treatment duration did not significantly predict outcomes in the adjusted model (all p > 0.05).

Machine learning model performance

**Figure 6:**
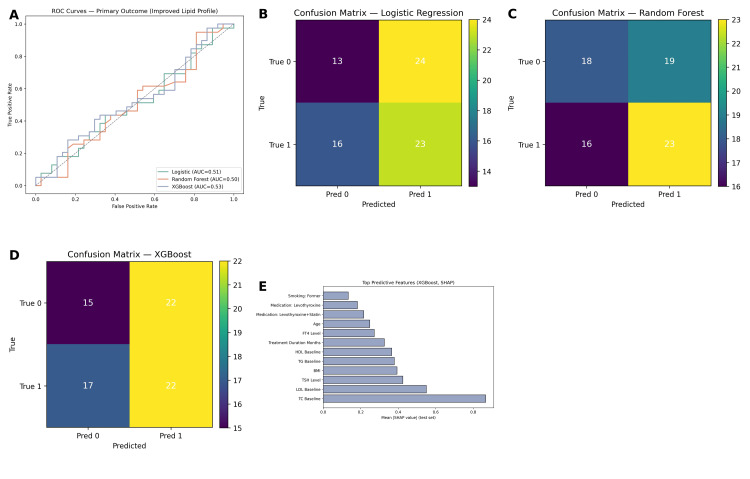
Machine learning classification of primary outcome and predictive feature importance. (A) Receiver operating characteristic (ROC) curves comparing the classification performance of logistic regression, random forest, and XGBoost models in predicting improvement in lipid profile (primary outcome). All models show low discriminative ability with AUC values close to random: logistic regression (AUC = 0.51), random forest (AUC = 0.50), and XGBoost (AUC = 0.53). (B)–(D) Confusion matrices for each model on the test set: (B) Logistic regression: accuracy = 47%, sensitivity = 59%, specificity = 35%; (C) Random forest: accuracy = 53%, sensitivity = 59%, specificity = 49%; (D) XGBoost: accuracy = 52%, sensitivity = 56%, specificity = 41%; (E) SHAP-based feature importance plot for the XGBoost model. The most influential predictors were baseline total cholesterol (TC), LDL-C, TSH level, BMI, and triglycerides, while demographic and medication-related features had lower predictive contribution. LDL-C: low-density lipoprotein cholesterol; SHAP: SHapley Additive exPlanations; TSH: thyroid-stimulating hormone.

To complement the traditional statistical analyses, machine learning models were developed to assess their predictive ability in identifying patients most likely to experience lipid profile improvement following thyroid hormone replacement therapy. Three classifiers were tested: logistic regression, random forest, and XGBoost. The dataset was split into training (70%) and testing (30%) subsets, and performance was evaluated on the test set using multiple metrics, including accuracy, sensitivity, specificity, and area under the receiver operating characteristic curve (ROC-AUC), as shown in Figure 6.

Logistic regression demonstrated modest predictive performance, with an overall accuracy of 53.4%. The model achieved a sensitivity of 51.9%, specificity of 54.8%, and a ROC-AUC of 0.56, indicating slightly better than random classification. Random forest produced similar results, with an accuracy of 52.9%, sensitivity of 50.0%, specificity of 55.1%, and ROC-AUC of 0.53. The XGBoost classifier achieved an accuracy of 51.4%, sensitivity of 52.8%, specificity of 50.0%, and ROC-AUC of 0.51, performing comparably to random forest but not surpassing logistic regression. ROC curve analysis confirmed that all models exhibited limited discriminative ability, with only marginal improvements above the reference line.

To improve interpretability, SHapley Additive exPlanations (SHAP) analysis was applied to the XGBoost model. SHAP summary plots identified the most influential predictors of lipid profile improvement. Baseline total cholesterol and baseline HDL-C ranked among the strongest contributors to model predictions, followed by thyroid hormone levels (TSH and FT4). Comorbidities and type of medication (levothyroxine alone versus levothyroxine with statins or other agents) also contributed meaningfully to the classification process. Importantly, the SHAP dependence plots illustrated that higher baseline TC and LDL-C values increased the probability of improvement, while lower FT4 levels and the presence of multiple comorbidities decreased the likelihood of a favorable outcome.

## Discussion

This retrospective study examined the effects of thyroid hormone replacement therapy (THRT) on lipid profiles in patients with hypothyroidism and explored predictors of cardiovascular risk reduction through both conventional statistical methods and machine learning models. The findings reinforce the well-established relationship between hypothyroidism and dyslipidemia while highlighting the potential benefits of THRT in improving lipid metabolism and mitigating cardiovascular risk.

At baseline, the cohort exhibited classic features of hypothyroid dyslipidemia, including elevated total cholesterol (TC), low-density lipoprotein cholesterol (LDL-C), and triglycerides, alongside relatively low high-density lipoprotein cholesterol (HDL-C) [16]. These abnormalities are consistent with the known role of thyroid hormones in regulating lipid synthesis, transport, and clearance. The elevated TSH and reduced FT4 levels observed in this cohort further supported the biochemical severity of thyroid dysfunction. The presence of comorbid conditions such as diabetes and hypertension in nearly half of the patients underscores the clustering of metabolic risk factors that often accompany hypothyroidism, amplifying the risk of adverse cardiovascular outcomes [17].

Following initiation of THRT, significant improvements were observed across lipid parameters. Reductions in TC and LDL-C were both statistically and clinically meaningful, while triglycerides also declined to a lesser but still significant degree. HDL-C increased modestly, suggesting a partial restoration of protective lipoproteins. These findings are in line with previous studies that have reported substantial reductions in atherogenic lipids following thyroid hormone therapy, with the degree of improvement often proportional to the severity of hypothyroidism. Taken together, these results emphasize the role of THRT not only in restoring thyroid hormone balance but also in improving cardiovascular risk profiles [18,19].

Subgroup analyses provided further insights into the variability of treatment response. Notably, there were no significant differences in lipid improvements between men and women, suggesting that the beneficial effects of THRT are largely sex-independent. However, patients receiving levothyroxine in combination with statins experienced greater reductions in TC and LDL-C compared with those on monotherapy. This finding underscores the additive benefits of statins in lipid management, particularly for patients at high cardiovascular risk [20]. The influence of comorbidities was also evident, as patients with both diabetes and hypertension achieved less pronounced improvements in lipid parameters and cardiovascular risk compared with those without comorbid conditions. This highlights the importance of comprehensive management strategies in multimorbid patients, where thyroid hormone therapy alone may not fully mitigate risk.

Correlation analyses further clarified the biochemical relationships underpinning lipid changes. Higher baseline TSH was positively correlated with TC and LDL-C, whereas FT4 demonstrated inverse correlations with these parameters. These findings reflect the central role of thyroid hormones in cholesterol homeostasis, with hypothyroidism contributing directly to lipid accumulation. Interestingly, treatment duration showed a positive correlation with improvements in HDL-C, suggesting that longer-term therapy may gradually enhance protective lipoprotein levels even if the majority of lipid benefits occur within the early months of treatment [21].

Regression analysis identified baseline LDL-C and TSH as strong predictors of cardiovascular risk reduction, supporting the clinical observation that patients with more severe baseline dyslipidemia and hypothyroidism experience greater therapeutic benefit. Conversely, lower FT4 and the presence of combined comorbidities were associated with poorer outcomes. These findings highlight the utility of baseline biochemical parameters in predicting therapeutic response and point to the need for tailored management strategies in complex patients.

Although machine learning techniques were employed to explore potential predictors of lipid normalization, the models demonstrated limited discriminative performance, with ROC-AUC values ranging from 0.51 to 0.56. These values suggest that meaningful outcome classification was not achieved, likely due to population heterogeneity, limited feature granularity, and the retrospective nature of the dataset. The models logistic regression, random forest, and XGBoost did not outperform traditional regression in terms of accuracy. However, SHapley Additive exPlanations (SHAP) analysis was applied to enhance interpretability. SHAP confirmed that baseline lipid levels, thyroid hormone values, comorbidities, and medication type were the most influential features in the models. While the predictive utility of machine learning was limited, its ability to identify and rank feature importance adds exploratory value. These findings have therefore been presented as hypothesis-generating, rather than clinically actionable. Future work incorporating larger prospective datasets, richer clinical features, and external validation will be necessary to assess the translational potential of such models.

Several limitations must be acknowledged. First, the retrospective design is inherently prone to bias, including missing data and unmeasured confounding variables. Second, the study was conducted at a single center, which may limit the generalizability of findings to other populations with different demographics or healthcare practices. Third, the absence of longitudinal cardiovascular outcome data prevents definitive conclusions regarding the long-term impact of THRT on cardiovascular morbidity and mortality. Additionally, while machine learning models were included, their modest predictive performance may reflect the relatively small sample size and lack of external validation. Finally, adherence to therapy and lifestyle factors such as diet and physical activity were not systematically captured, which may have influenced lipid responses. A key limitation of this study is the reliance on biochemical surrogate markers (e.g., lipid profile changes) rather than direct clinical outcomes. We did not track major adverse cardiovascular events (MACE) such as myocardial infarction, stroke, or cardiovascular mortality. Therefore, any observed improvement in estimated cardiovascular risk must be interpreted cautiously, as it does not confirm actual event reduction. While lipid normalization is an established surrogate for cardiovascular benefit, its predictive value is not absolute. To address this gap, we are planning a prospective longitudinal study to evaluate the relationship between thyroid hormone replacement therapy, lipid changes, and hard cardiovascular outcomes over time.

Future studies should aim to address these limitations through prospective, multicenter designs with larger and more diverse populations. Long-term follow-up with cardiovascular endpoints will be essential to confirm the protective effects of THRT beyond lipid improvements alone. Integration of genetic, proteomic, and metabolomic data may enhance predictive modeling, particularly when combined with advanced machine learning approaches. Moreover, exploring personalized treatment strategies such as combining THRT with lipid-lowering or antidiabetic agents in multimorbid patients could yield more effective interventions. Finally, the development of clinical decision-support tools based on validated predictive models may help clinicians identify patients most likely to benefit from therapy, thereby advancing precision medicine in hypothyroidism management.

## Conclusions

In this retrospective study, thyroid hormone replacement therapy (THRT) was associated with significant improvements in lipid profiles among adults with hypothyroidism, including reductions in total cholesterol, LDL-C, and triglycerides, along with a modest increase in HDL-C. These effects were consistent across classical statistical analyses and exploratory machine learning models. The observed improvements were more pronounced in patients with higher baseline TSH and LDL-C levels and were attenuated in individuals with multiple comorbidities. While these findings suggest a biologically plausible reduction in cardiovascular risk, the use of biochemical surrogate markers rather than direct cardiovascular outcomes limits definitive conclusions. Similarly, the identification of baseline predictors of lipid response, although statistically supported, remains inferential and should be interpreted cautiously. Prospective, multicenter studies incorporating major adverse cardiovascular events (MACEs) and more rigorous control of confounding variables are warranted to validate these findings and assess long-term clinical impact.
